# Mycobacteria Counteract a TLR-Mediated Nitrosative Defense Mechanism in a Zebrafish Infection Model

**DOI:** 10.1371/journal.pone.0100928

**Published:** 2014-06-26

**Authors:** Philip M. Elks, Michiel van der Vaart, Vincent van Hensbergen, Esther Schutz, Michael J. Redd, Emi Murayama, Herman P. Spaink, Annemarie H. Meijer

**Affiliations:** 1 Institute of Biology, Leiden University, Leiden, South Holland, The Netherlands; 2 The Bateson Centre, University of Sheffield, Sheffield, South Yorkshire, United Kingdom; 3 Department of Infection and Immunity, University of Sheffield, Sheffield, South Yorkshire, United Kingdom; 4 Department of Oncological Sciences, University Of Utah, Salt Lake City, Utah, United States of America; 5 Unité Macrophages et Développement de l’Immunité, Institut Pasteur, Paris, France; 6 Centre National de la Recherche Scientifique-Unité de Recherche Associée 2578, Institut Pasteur, Paris, France; Karlsruhe Institute of Technology, Germany

## Abstract

Pulmonary tuberculosis (TB), caused by the intracellular bacterial pathogen *Mycobacterium tuberculosis* (Mtb), is a major world health problem. The production of reactive nitrogen species (RNS) is a potent cytostatic and cytotoxic defense mechanism against intracellular pathogens. Nevertheless, the protective role of RNS during Mtb infection remains controversial. Here we use an anti-nitrotyrosine antibody as a readout to study nitration output by the zebrafish host during early mycobacterial pathogenesis. We found that recognition of *Mycobacterium marinum*, a close relative of Mtb, was sufficient to induce a nitrosative defense mechanism in a manner dependent on MyD88, the central adaptor protein in Toll like receptor (TLR) mediated pathogen recognition. However, this host response was attenuated by mycobacteria via a virulence mechanism independent of the well-characterized RD1 virulence locus. Our results indicate a mechanism of pathogenic mycobacteria to circumvent host defense in vivo. Shifting the balance of host-pathogen interactions in favor of the host by targeting this virulence mechanism may help to alleviate the problem of infection with Mtb strains that are resistant to multiple drug treatments.

## Introduction

Pulmonary tuberculosis (TB) is a common and often lethal infectious disease caused by the pathogenic bacterium *Mycobacterium tuberculosis* (Mtb), which represents a major global health threat. The current high incidence and associated mortality worldwide is partly explained by the increasing occurrence of multi-drug resistant Mtb strains. This makes TB a key priority for infectious disease research, since understanding of the host-pathogen interactions during Mtb pathogenesis is necessary to develop novel therapeutic strategies [1], [2]. Mycobacteria phagocytosed by macrophages are able to evade the majority of leukocyte bacterial killing mechanisms and create a niche for themselves in which they can survive and proliferate [3]. Recruitment of macrophages, neutrophils, and T-cells to the primary infected cells leads to the formation of highly organized structures known as granulomas [4], [5]. During latent TB infection, a condition estimated to affect one-third of the human population, Mtb can persist for many years within granulomas before its reactivation and dissemination induces an active disease state [6].

The combined efforts of the innate and adaptive arms of the immune system are relatively efficient in containing Mtb infection in immunocompetent individuals. It is estimated that only 5–10 out of a 100 individuals newly infected with Mtb develop active TB during their lifetime [7]. One of the most effective host-defense mechanisms against intracellular pathogens is the production of reactive oxygen species (ROS) and reactive nitrogen species (RNS) [8], [9]. The production of ROS, such as hydrogen peroxide (H_2_O_2_), superoxide anion (O_2_^−^), and hydroxyl radical (OH^•^), is catalyzed by the NADPH-oxidase complex during respiratory burst [8]. In vertebrates nitric oxide (NO) is formed when the guanidine nitrogen of L-arginine is oxidized by nitric oxide synthases (NOS). There are two constitutively expressed NOS enzymes, neuronal NOS (nNOS or NOS1) and endothelial NOS (eNOS or NOS3), and an inducible NOS enzyme (iNOS or NOS2). Regulation of *iNOS* transcription plays an important role in the inflammatory and infection response and many cells of the immune system are capable of producing NO [10].

Although a mutation in the components of the NADPH-complex increased susceptibility to Mtb [11]–[13], ROS production itself was found to be relatively ineffective at killing Mtb [14]. In contrast, exposure to low concentrations of NO is cytotoxic to mycobacteria, killing more than 99% of Mtb in culture [15]. However, a significant part of the cytostatic and cytotoxic antimicrobial effects of NO in vivo are attributed to RNS, the various reaction products of nitric oxide radicals (NO^•^) [16]. The reaction of NO radicals with oxygen radicals (produced during the respiratory burst) results in the generation of peroxynitrite (ONOO^−^), a potent RNS reaction that causes lipid peroxidation, DNA damage, oxidation of thiols, and nitration of tyrosine residues [17], [18]. Tyrosine nitration can also be catalyzed by the neutrophil-specific enzyme myeloperoxidase (Mpx), using H_2_O_2_ and NO_2_^−^ as substrates [19]. The bactericidal activity of peroxynitrite has been demonstrated against *Salmonella enterica* serovar Typhimurium [20], and *Escherichia coli* [21]. Peroxynitrite is also required for the fungicidal activity of macrophages infected with *Candida albicans* [22].

An essential role for NO in the killing of Mtb by mononuclear phagocytes was demonstrated in a murine TB model [23], [24]. Furthermore iNOS deficient mice were highly susceptible to Mtb infection [11], [25], [26]. In contrast, the cytostatic and cytotoxic role of NO during Mtb infection in humans is debated, since early mycobacteriostatic activity of human macrophages was found to be independent of NO [27], [28]. Nevertheless, there is an increasing amount of evidence that NO produced by macrophages and epithelial cells also provides protection against Mtb in humans [26], [29]–[33]. In agreement with this hypothesis, polymorphisms in the gene for iNOS have been associated with susceptibility to TB in different human populations [34]. The conflicting results on the role of NO and iNOS in defense against mycobacterial infection may, in part, be explained by the range of mechanisms utilized by Mtb to counteract ROS and RNS produced by the host [35]. Oxidative and nitrosative stresses induce a transcriptional response in *Mtb* that can lead to direct scavenging of reactive species, repair and protection of bacterial proteins as well as DNA [35], [36].

The natural fish pathogen *Mycobacterium marinum* (Mm) is a close relative of human Mtb and displays similar pathogenesis to the human disease, including the formation of caseating granulomas and development of latency [37]–[40]. The zebrafish embryo model, which allows for detailed observation of host-pathogen interactions, is well-established for the in vivo study of host signaling and mycobacterial virulence determinants during early stages of TB disease [41]–[43]. In this study we use an antibody to detect nitration of tyrosine residues as readout for the nitrosative response of the zebrafish host to infection with Mm [44]–[46]. We found that Mm infection led to an increase of nitrosative stress in neutrophils which was dependent on the function of MyD88, the universal signal adaptor protein utilized by Toll-like receptors (TLRs) to initiate innate immunity upon detection of microbial ligands [47]. During the pathogenesis of infection, this host response was attenuated by live Mm via a virulence mechanism independent of the well-characterized mycobacterial RD1 virulence locus [3]. Our results shed light on the intriguing host-pathogen interactions that occur during the onset of mycobacterial disease and add to our understanding of RNS as anti-mycobacterial defense mechanisms in vivo.

## Materials and Methods

### Ethics Statement

Zebrafish lines were handled in compliance with the local animal welfare regulations and maintained according to standard protocols (zfin.org). The breeding of adult fish was approved by the local animal welfare committee (DEC) of the University of Leiden and adhered to the international guidelines specified by the EU Animal Protection Directive 2010/63/EU. Adult zebrafish were not sacrificed for this study. All experiments in this study were performed on embryos/larvae at 48, 72 and 120 hours post fertilization (hpf) before the free-feeding stage and did not fall under animal experimentation law according to the EU Animal Protection Directive 2010/63/EU. Embryos were anaesthetized with 0.02% Tricaine (MS-222) and subsequently euthanized with 4% paraformaldehyde at 2, 4, and 5 days post-fertilization for immuno-histochemical and microscopy analysis.

### Fish husbandry

Zebrafish strains (wildtype strain used was AB/TL) were maintained according to standard protocols. Transgenic and mutant strains used were *Tg(mpx:GFP)i114* [48], *Tg(lyz-DsRED2)nz50* [49], *myd88−/−hu3568*/*Tg(mpx:GFP)i114* [50], and *mpx−/−NL144*/*Tg(mpx:GFP)i114*. The *mpx−/−NL144* “spotless” mutant line harbors a C to T point mutation at nucleotide position 1126 of the mpx RefSeq mRNA sequence (NM_212779) resulting in a premature stop codon (M. R., E. M., W. Horsley, N. Trede, and P. Herbomel, unpublished data). Adult fish were maintained on a 14 hour light and 10 hour dark cycle at 28°C in accordance with local animal welfare regulations.

### Bacterial culture preparation

Infection experiments were performed using *M. marinum* strain M (ATCC #BAA-535) [51] and its mutant derivative ΔRD1 [52], both containing the pSMT3-mCherry vector [53]. Liquid cultures were prepared from bacterial plates as previously described [53] with 50 µg/ml hygromycin. Injection inoculum was prepared from overnight liquid cultures (at OD600<1) by washing three times in sterile 0.05% Tween80/PBS solution (BD Difco, Breda, The Netherlands), assessing optical density at 600 nm and resuspending in a 2% polyvinylpyrrolidone40 (PVP40) solution (CalBiochem, Merck Millipore, Nottingham, UK) in PBS [54].

### Intravenous injection of bacteria into one-day old embryos

Injection of bacteria was performed into the blood-forming region above the caudal vein at 28 hpf as previously described [54]. An inoculum volume of 1 nl and 100 colony-forming units (CFU) of bacteria were injected. CFU was checked by plating out the injection volume.

### DAF-FM DA Staining

At the timepoint of infection 5 µM DAF-FM DA in embryo water was applied to a Petri dish of around 30 embryos as previously described [55], [56]. DAF-FM DA was washed off using embryo water at 1 dpi and imaged using confocal microscopy.

### Stereoimaging and bacterial pixel quantification

Embryos were imaged at 4 dpi on a Leica MZ16FA Fluorescence Stereo Microscope. Brightfield and fluorescence images were generated with a Leica DC500 (DFC420C) camera. Bacterial loads were analysed using dedicated pixel counting software as previously described [42].

### Antibody detection of Nitrotyrosine, iNOS and TSA staining

Larvae were fixed in 4% paraformaldehyde in PBS overnight at 4°C and nitrotyrosine levels were immune-labelled using a rabbit polyclonal anti-nitrotyrosine antibody (Merck Millipore 06-284, MA, USA) at a 1∶200 dilution of primary antibody, and were detected using an Alexa Fluor (Invitrogen Life Technologies, NY, USA) secondary antibody. Mpx activity was labelled with TSA (TSAplus kit, Fluorescence Systems, Perkin Elmer Inc., Waltham, MA, USA) as previously described [57], [58].

For iNOS antibody staining, larvae were fixed in 4% paraformaldehyde in PBS overnight at 4°C and iNOS was immuno-labeled with a rabbit polyclonal iNOS antibody (BD Biosciences, Breda, The Netherlands) as previously described [59]. Detection was with goat anti-rabbit HRP-conjugated antibody (Abcam, 1∶500 dilution) and Cy3Plus TSA kit (Perkin-Elmer, Waltham, MA, USA).

### Confocal microscopy and quantification of corrected cell fluorescence of anti-nitrotyrosine levels

Embryos were imaged at 1 dpi, in the presence or absence of infection, embedded in 1% low melting point agarose (Sigma-Aldrich, St. Louis, MO, USA) and transferred to a Leica DMIRBE inverted microscope with a Leica SP1 confocal scanhead for imaging with 40 or 63 times lenses. For quantification purposes acquisition settings and area of imaging (in the caudal vein region) were kept the same across the groups. Corrected total cell fluorescence was calculated for each immune-stained cell using Image J measurements as previously described [46], [60]. Single cells (neutrophils) were selected for by the expression of GFP from the *Tg(mpx:GFP)i114* line. These measurements assess the cell fluorescence of each individual immune cell, corrected for cell size and background fluorescence of the image.

### Inhibition of il8/Cxcr2 signaling

Il8 signaling was inhibited using a previously described splice blocking morpholino (an injection mixture of *cxcl8-l1* E1/I1 and *cxcl8-l2* E1/I1) [61]. Cxcr2 was inhibited using the selective nonpeptide inhibitor SB225002 (Tocris Bioscience, Bristol, UK), as previously described [61], at 4 hours before infection until 24 hpi.

### RNA injections of dominant active *hif-1αb*

Embryos were injected with dominant active *hif-1αb* (ZFIN: *hif1ab*) RNA at the one cell stage as previously described [46].

### Statistical analysis

All data were analysed (Prism 5.0, GraphPad Software, San Diego, CA, USA) using unpaired, two-tailed t-tests for comparisons between two groups and one-way ANOVA (with Bonferroni post-test adjustment) for multiple group comparisons.

## Results

### Neutrophils display basal levels of tyrosine nitration dependent on myeloperoxidase activity

**Figure 1:**
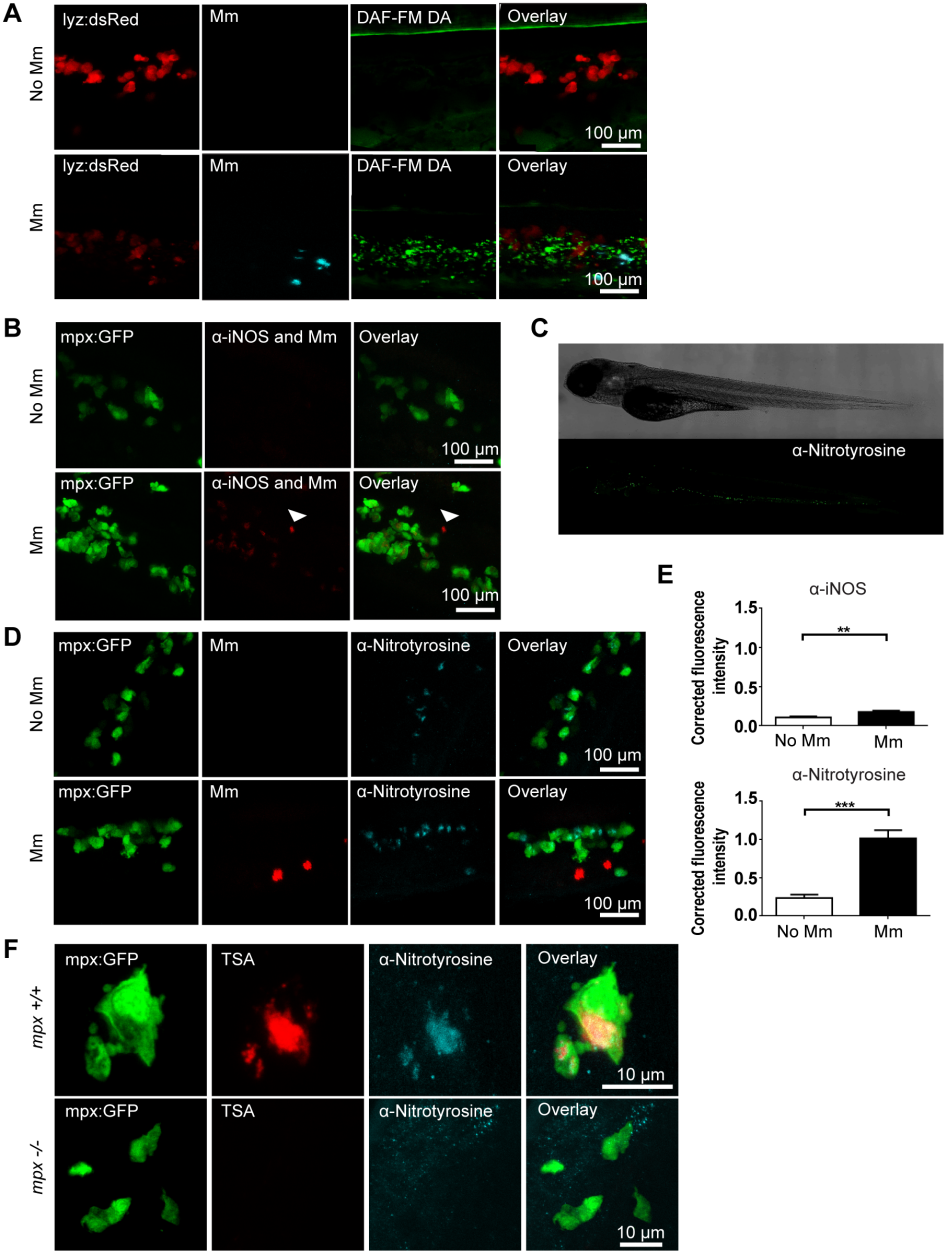
Tyrosine nitration is a quantifiable measure of nitration output during early Mm infection. (A) Confocal micrographs of DAF-FM DA stained embryos at 1 day post infection (dpi) in the caudal vein region. Neutrophils are shown by *lyz* driven dsRed. Upper panels show an example of an uninfected embryo while lower panels show an infected embryo. N.b. The bright stripe of DAF-FM DA staining in the upper panels is the notochord, which has variable brightness when focusing on the caudal vein region due to differences in embryo mounting. (B) Confocal micrographs of 1 dpi embryos stained with iNOS antibody (dim red) when infected with Mm (bright red, white arrow) or non-infected. Neutrophils are shown by *mpx*:GFP. (C) Fluorescence micrographs of confocal images stitched together to show the location of anti-nitrotyrosine staining in a whole-mount wild type zebrafish embryo of 3 dpf. (D) Confocal fluorescence micrographs of 1 dpi embryos showing the co-localization of *mpx:*GFP and anti-nitrotyrosine antibody staining in the absence or presence of Mm infection. (E) Corrected fluorescence intensity measurements of antibody stainings in the absence or presence of Mm. Upper panel shows the levels for the iNOS antibody shown in (B). Data shown are mean ± SEM, n = 45 cells from 15 embryos combined from 3 independent experiments. Lower panel shows the levels for the anti-nitrotyrosine antibody shown in D). Data shown are mean ± SEM, n = 58–99 cells from 15 embryos combined from 3 independent experiments. (F) Confocal micrographs showing co-localization of nitrotyrosine with compartments of neutrophils with myeloperoxidase activity (shown by TSA staining, in the absence of infection at 2 dpf. In the *mpx^−/−^* mutant there is no myeloperoxidase activity and a corresponding decrease in nitrotyrosine.

Detection of iNOS in the zebrafish model has been previously reported, however detection measures are often limited by either poor specificity for different types of NOS, or by relatively low levels of expression [56], [62]. In order to detect whether infection by Mm increased NO output we employed 3 different methods of detection. DAF-FM DA is a vital stain that detects NO produced by all NOS isoforms, and can be added to the embryo water during the first hours of infection [56]. We observed that staining of DAF-FM DA increased after Mm infection (figure 1A). However, DAF-FM DA has the disadvantage of being a marker for NO produced by all NOS isoforms, therefore it is difficult to assess in a quantitative manner the increase in iNOS output specifically. An alternative method is to utilize an antibody against iNOS itself [62] to detect iNOS protein levels. This has the advantage of specifically labeling iNOS, but levels of iNOS in leukocytes is low on a whole-organism setting, and the wholemount antibody staining requires amplification using TSA therefore reducing the potential to accurately quantify the amount of iNOS produced, although an increase was observed after Mm infection (figure 1B and E).

Tyrosine nitration is a hallmark of nitrosative stress caused by ROS and RNS produced by macrophages and neutrophils [44]. An antibody against nitrated tyrosine residues has been successfully used in common carp (*Cyprinus carpio*) to study the differential contribution of macrophages and neutrophils to nitrosative stress in response to a parasitic infection [44]. We have previously used a similar approach as readout for nitrosative stress induced by mycobacterial infection and Hif-1α stabilization in zebrafish embryos [45], [46]. In the absence of infection, tyrosine nitration was mainly detectable in a cellular pattern in the caudal hematopoietic tissue, displaying a similar distribution to that of neutrophils (figure 1C). Indeed, using the mpx:GFP transgenic line, which labels all neutrophils with GFP [48], we determined that the basal levels of anti-nitrotyrosine staining colocalized with neutrophils in the absence of infection (Figure 1D). Neutrophils display high basal levels of tyrosine nitration, since the neutrophil-specific peroxidase, Mpx (myeloperoxidase), catalyzes tissue nitration without peroxynitrite present [44], [46]. In Mm infected embryos anti-nitrotyrosine was mainly co-localized with neutrophils both at early stages of infection and in granulomas (figure 1D and figure S1). Upon Mm infection levels of anti-nitrotyrosine staining were significantly increased compared to uninfected controls (figure 1D and E) at 1 dpi. Mpx activity can be visualized in zebrafish embryos using an immunohistochemical peroxidase activity detection (TSA) protocol [57]. The cellular localization of Mpx co-localized with anti-nitrotyrosine staining in neutrophils of wild type embryos, while both neutrophil TSA and anti-nitrotyrosine staining were undetectable in embryos mutated in Mpx (Figure 1F). These data confirm that the basal levels of nitrotyrosine in neutrophils is dependent on Mpx activity.

### Nitrotyrosine production is an early host response to *Mycobacteria marinum* infection

**Figure 2:**
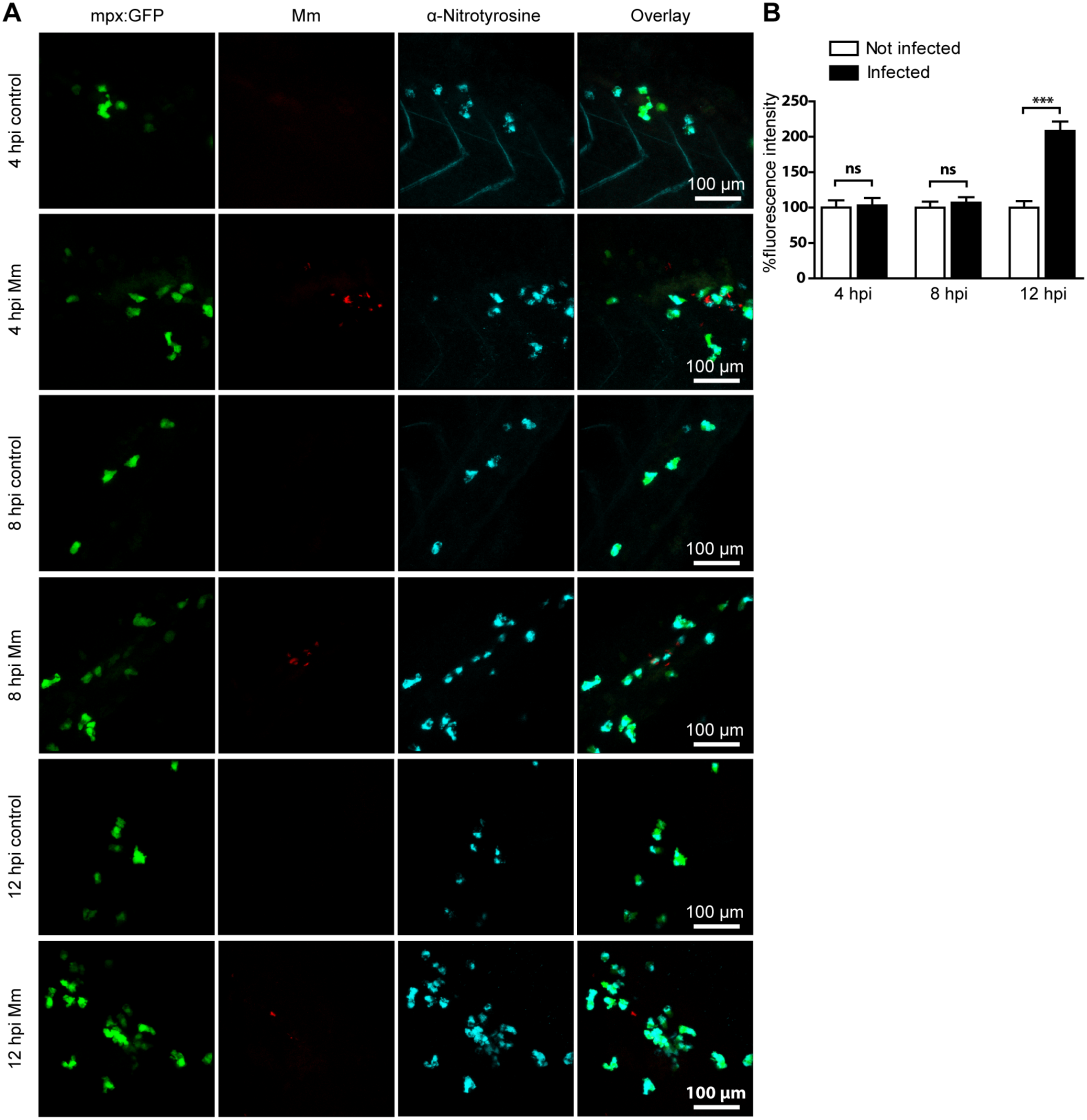
Mm infection increased tyrosine nitration levels at 12 hpi. (A) Example fluorescence confocal z-stacks of the caudal vein region of embryos stained with anti-nitrotyrosine antibody, imaged at 4, 8 or 12 hpi in the presence or absence of Mm infection. (B) Corrected fluorescence intensity levels of anti-nitrotyrosine antibody confocal z-stacks of equal size at 4, 8 or 12 hours after Mm infection relative to the control group per time point. Data shown are mean ± SEM, n = 50–85 cells from 15 embryos combined from 3 independent experiments.

**Figure 3:**
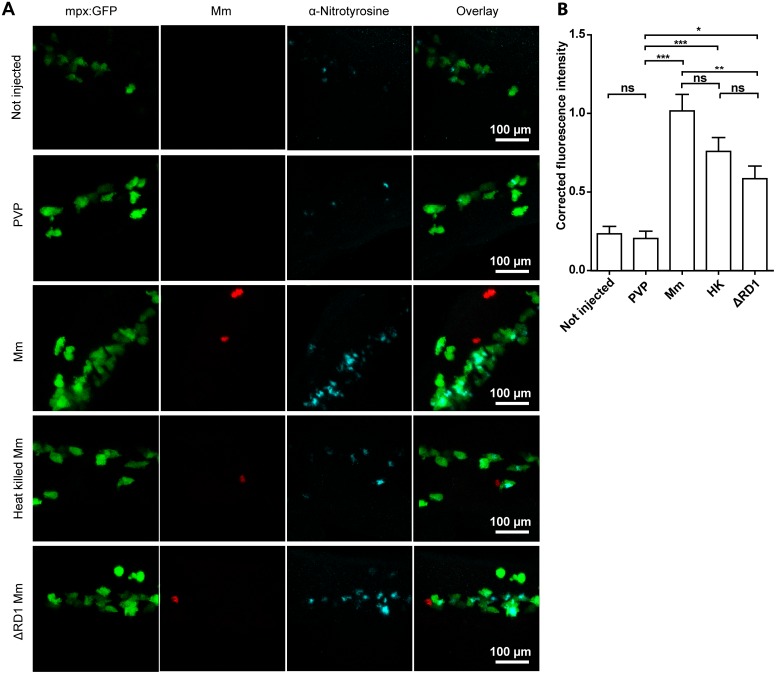
Injection with live, heat-killed or ΔRD1 Mm increased tyrosine nitration levels. (A) Example fluorescence confocal z-stacks of the caudal vein region of embryos stained with anti-nitrotyrosine antibody, imaged 1 day after injection with live, heat killed or ΔRD1 Mm in comparison with uninjected embryos or embryos injected with 2% PVP carrier solution. (B) Corrected fluorescence intensity levels of anti-nitrotyrosine antibody confocal z-stacks of equal size 1 day after injection with live, heat killed or ΔRD1 Mm. Data shown are mean ± SEM, n = 58–99 cells from 15 embryos combined from 3 independent experiments.

Mm are rapidly phagocytosed by macrophages when injected into the circulation of zebrafish embryos [62]. We have previously demonstrated that tyrosine nitration levels were elevated in neutrophils one day post infection (dpi) with Mm and that this response could be inhibited by a morpholino against *nos2a*, one of the zebrafish genes for iNOS [46]. To better understand the dynamics of this process, we looked at nitrotyrosine levels in neutrophils at earlier time points after infection with Mm. At 4 and 8 hours post infection (hpi) levels of anti-nitrotyrosine staining in neutrophils of infected embryos were not significantly different to uninfected controls. However, by 12 hpi the levels of tyrosine nitration in neutrophils were significantly higher in infected embryos, although the neutrophils themselves did not necessarily contain bacteria (figure 2A and B). The lag time between the onset of infection and detectable increased tyrosine nitration is consistent with infection-induced RNS production first requiring de novo synthesis of iNOS [26]. Furthermore, the lag time may indicate that infected macrophages first must signal to bystander neutrophils before the iNOS response is initiated. Increased nitration levels in neutrophils were observed after injection with live Mm, heat-killed Mm, or Mm with a deleted RD1 (ΔRD1) locus (figure 3A and B, figure S2). The RD1 virulence locus of pathogenic Mm contains genes for the ESX-1 secretion system and secreted factors required for survival and replication inside macrophages [63], [64]. These results demonstrate that the presence of Mm is sufficient to induce a leukocyte RNS response shortly after encountering bacteria, independent of its infectivity.

### Mm induced tyrosine nitration is independent of Il8/Cxcr2 signaling

**Figure 4:**
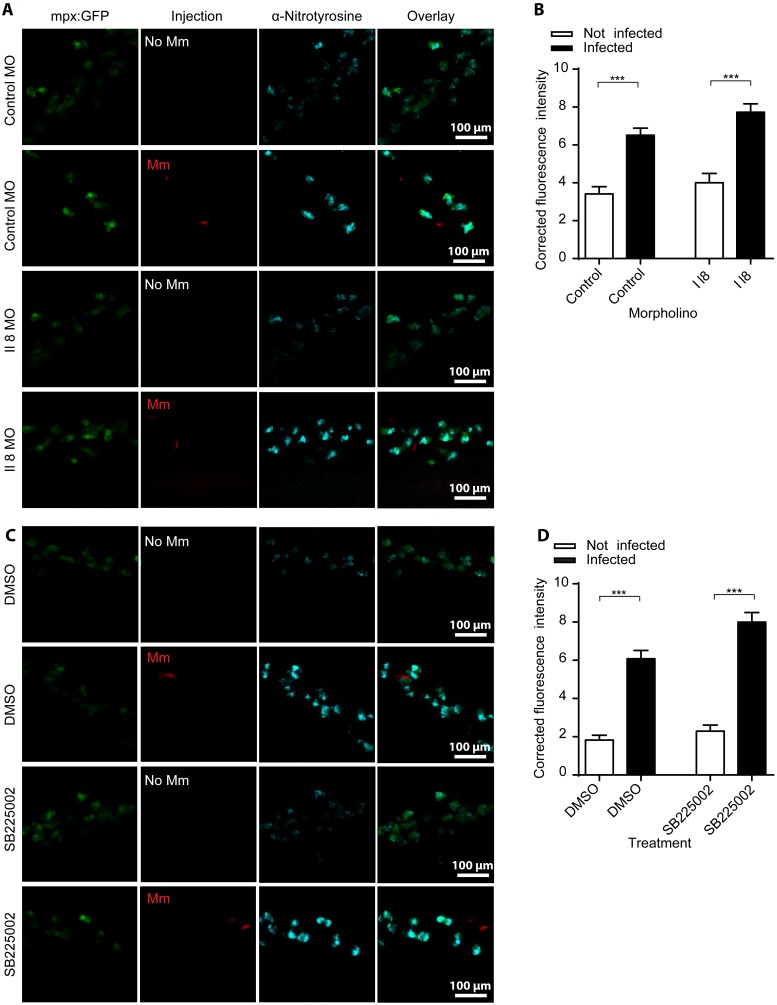
Increased nitrotyrosine levels in neutrophils post-infection is independent of Il8/Cxcr2 signaling. (A) Example fluorescent confocal micrographs of the caudal vein region stained with anti-nitrotyrosine at 1 dpi, following injection of either the standard control morpholino or the *il8* splice blocking morpholino at the 1-cell stage. Larvae shown are in the presence or absence of Mm, as indicated in the panels. (B) Corrected fluorescence intensity levels of anti-nitrotyrosine antibody confocal z-stacks of equal size 1 day after injection of control or *il8* morpholino. Data shown are mean ± SEM, n = 90 cells from 15 embryos combined from 3 independent experiments. (C) Example fluorescent confocal micrographs of the caudal vein region stained with anti-nitrotyrosine at 1 dpi, following treatment with the Cxcr2 inhibitor SB225002 or DMSO control. Larvae shown are in the presence or absence of Mm, as indicated in the panels. (D) Corrected fluorescence intensity levels of anti-nitrotyrosine antibody confocal z-stacks of equal size 1 day after treatment of DMSO or SB225002. Data shown are mean ± SEM, n = 90 cells from 15 embryos combined from 3 independent experiments.

After intravenous Mm infection it is primarily the macrophages that phagocytose the bacteria, and yet the upregulation of nitrotyrosine levels is found mainly in neutrophils. This upregulation of neutrophil nitrotyrosine is a systemic neutrophil response, rather than localized around areas of infection, therefore we postulated that chemokine signaling would be a likely candidate responsible for transduction of the signal from infected macrophages to neutrophils. Interleukin 8 (IL8) is a well characterized neutrophil attractant that is released by macrophages and epithelial/endothelial cells [65]. The receptors for IL8 include the chemokine receptors CXCR1 and CXCR2. CXCR2 is expressed on the cell surface of neutrophils and is the best characterized receptor for IL8’s role as a chemoattractant [66]. Furthermore, it has been shown that the zebrafish ortholog of IL8 can induce systemic activation of neutrophil motility through Cxcr2 signaling [67]. We therefore hypothesized that Il8/Cxcr2 signaling between infected macrophages and neutrophils may be a putative mechanism activating iNOS activity in neutrophils. To investigate this hypothesis, Il8 signal was blocked using a previously characterized Il8 splice blocking morpholino [61]. Blocking of Il8 did not lead to a loss or decrease of anti-nitrotyrosine staining after Mm infection (Figure 4A and B). Similarly, the Cxcr2 antagonist SB225002 caused no loss or decrease in anti-nitrotyrosine signal accumulation after Mm infection (figure 4C and D). These results rule out a role for the Il8/Cxcr2 signaling axis in the increase in neutrophil nitrotyrosine during Mm infection.

### TLR/IL1R-MyD88 signaling is required for Mm induced tyrosine nitration

**Figure 5:**
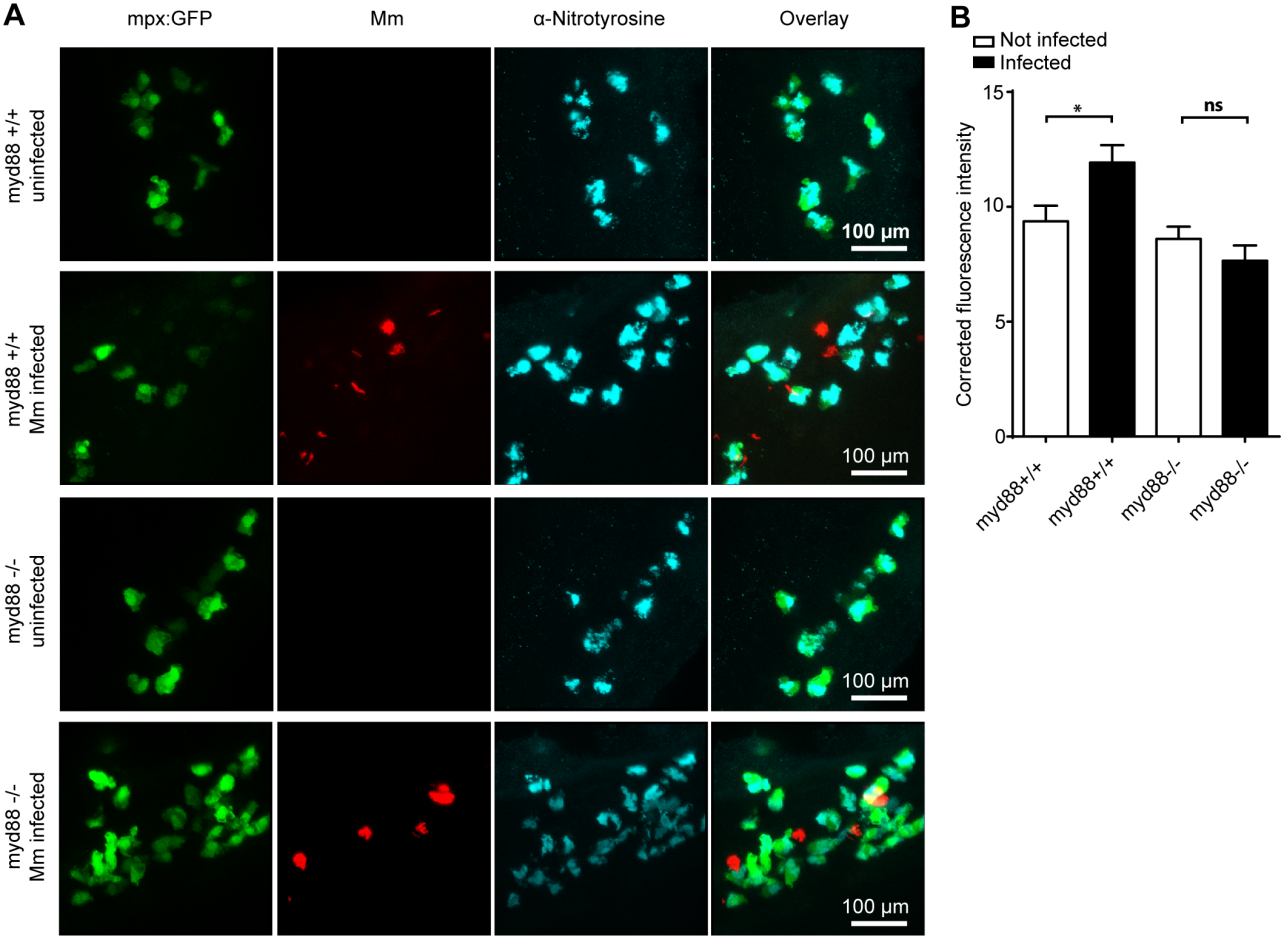
Increased tyrosine nitration following Mm infection is dependent on MyD88. (A) Fluorescent micrographs of confocal images showing the overlap between *mpx:GFP* and anti-nitrotyrosine staining in *myd88^+/+^* or *myd88^−/−^* embryos. (B) Example fluorescence confocal z-stacks of the caudal vein region of *myd88^+/+^* or *myd88^−/−^* embryos stained with anti-nitrotyrosine antibody, imaged at 1 dpi in the presence or absence of Mm infection. (C) Corrected fluorescence intensity levels of anti-nitrotyrosine antibody confocal z-stacks of equal size 1 day after injection with *Mm* in *myd88^+/+^* or *myd88^−/−^* embryos. Data shown are mean ± SEM, n = 30 cells from 5 embryos representative of 2 independent experiments.

Pattern recognition receptors, including Toll-like receptors (TLRs), can recognize pathogen associated molecular patterns (PAMPs) present on the exterior of mycobacteria and initiate an appropriate immune response, which can be further amplified by signaling through cytokine receptors such as the Interleukin 1 receptor (IL1R) [45], [68]. Stimulation of macrophages with TLR ligands increased the expression of *iNOS* in vitro [69], [70], and we have previously shown that a zebrafish mutant for the important TLR/IL1R signaling adaptor MyD88 (*myd88^−/−^*) was more susceptible to Mm infection [50]. The basal level of anti-nitrotyrosine staining in neutrophils was unchanged in uninfected *myd88^−/−^* embryos compared to *myd88^+/+^* embryos (figure 5A), indicating that nitration levels from myeloperoxidase are MyD88-independent. Anti-nitrotyrosine staining was significantly increased in infected *myd88^+/+^* embryos, whereas infected *myd88^−/−^* embryos did not show an increase compared to uninfected controls (figure 5A and B). These findings are consistent with Mm induced tyrosine nitration being dependent on MyD88-mediated signaling, most likely following TLR recognition of the pathogen.

### Neutrophil nitrotyrosine levels are depleted during Mm pathogenesis in a live-bacteria dependent mechanism

**Figure 6:**
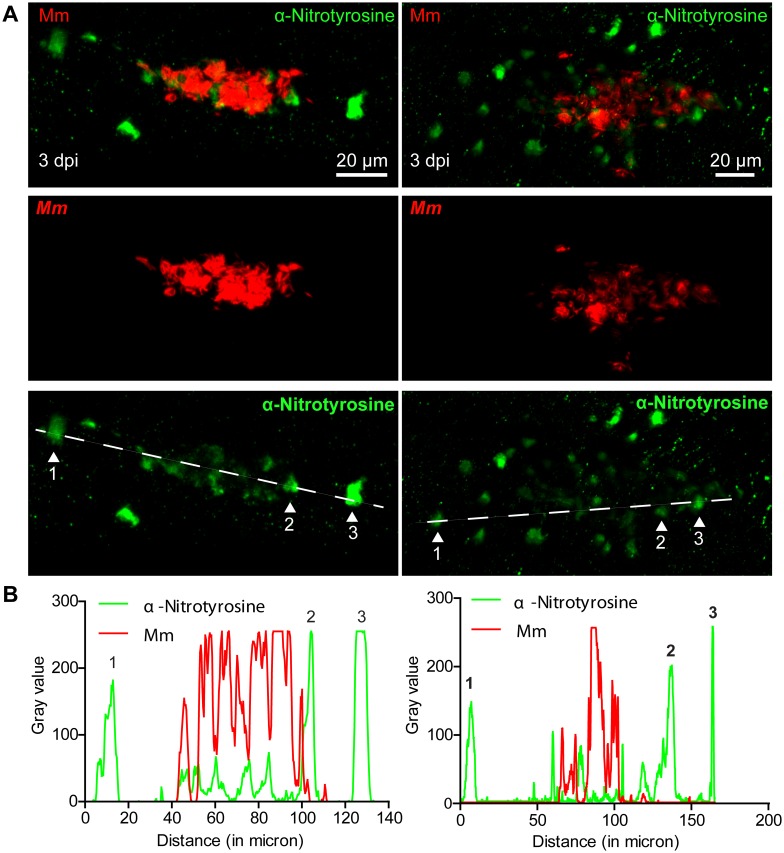
Nitrotyrosine levels are lower in the center of mycobacterial granulomas in zebrafish embryos. (A) Example fluorescence confocal micrographs of anti-nitrotyrosine staining performed on 3 dpi granuloma structures in wild type embryos infected with Mm showing a single Z-plane. (B) Gray values of the bacterial and anti-nitrotyrosine fluorescence signals measured along a straight line through the center of the granuloma along the longest axis of the granuloma (see white dashed lines in (A)), intensity of the fluorescent signal was measured using ImageJ. Numbered peaks in the graphs correspond to numbered patches of tyrosine nitration in (A).

We evaluated tyrosine nitration at later stages of Mm pathogenesis by performing anti-nitrotyrosine staining on zebrafish larvae with early granuloma structures. At 3 days post infection (dpi) anti-nitrotyrosine staining co-localized with, or was in proximity of, larval granulomas, demonstrating that nitrosative defense mechanisms remain active at this later time point of pathogenesis (figure 6A). Interestingly, we observed that the anti-nitrotyrosine staining in cells near the center of heavily infected areas was less intense than the staining in cells at the periphery of these areas, or in their direct proximity (figure 6B). The same phenomenon was observed for larger and more organized Mm-containing granulomas at 5 dpi (figure S3). These data are consistent with a decrease in neutrophil tyrosine nitration, as the majority of cells in the granuloma with anti-nitrotyrosine staining co-localise with myeloperoxidase (figure S1). Thus, while Mm infection initially increased nitrotyrosine levels of infected and non-infected neutrophils, these data are consistent with host RNS response being reduced in developing granulomas. These observations suggested that Mm might counteract the host’s nitrosative defense mechanism.

**Figure 7:**
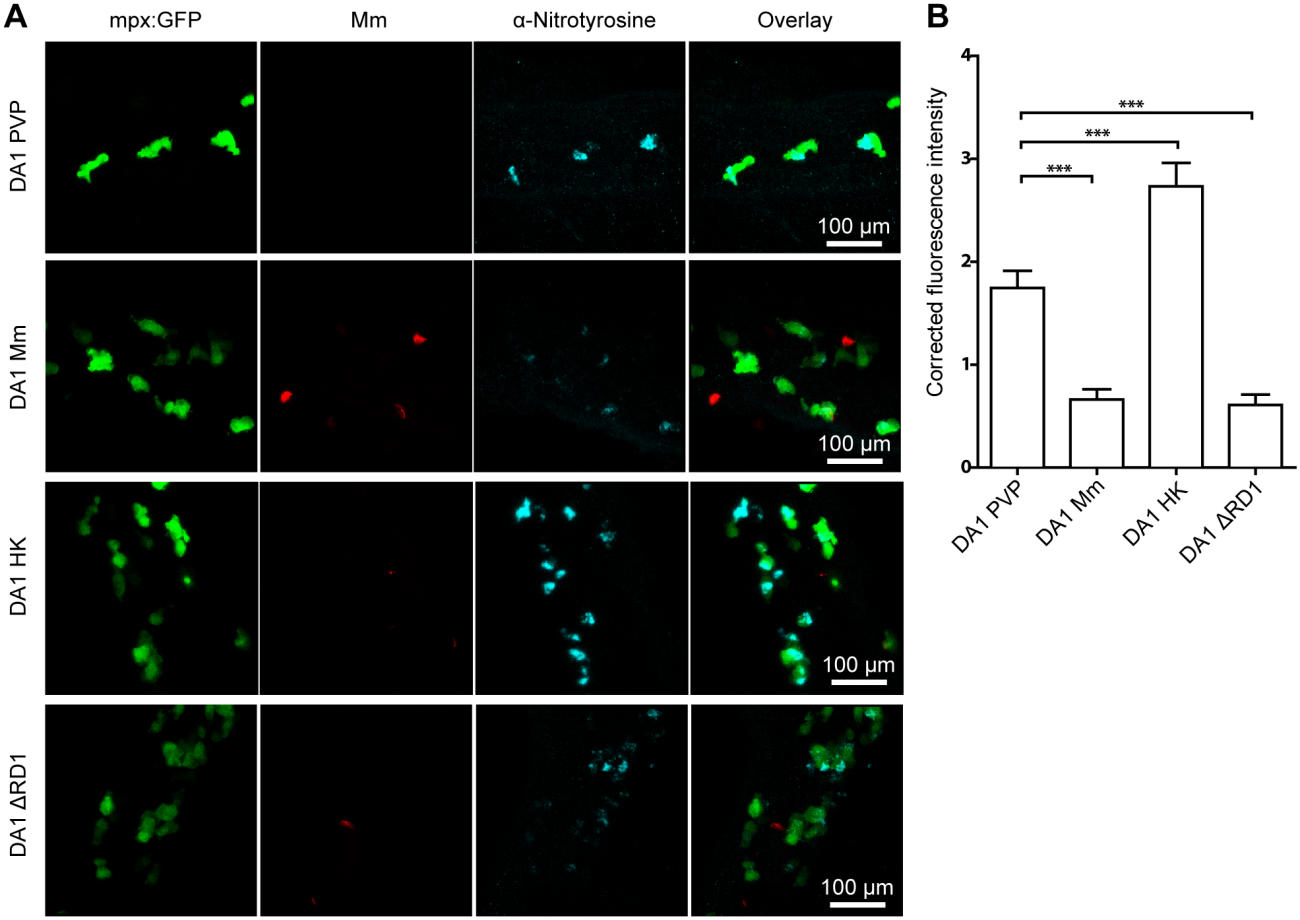
High levels of neutrophil tyrosine nitration are attenuated by live wild type orΔRD1 Mm, but not heat killed Mm. (A) Example fluorescence confocal z-stacks of the caudal vein region of dominant active *hif-1α* (DA1) mRNA injected embryos stained with anti-nitrotyrosine antibody, imaged 1 day after injection with live, heat killed or ΔRD1 Mm. (B) Corrected fluorescence intensity levels of anti-nitrotyrosine antibody confocal z-stacks of equal size on dominant active *hif-1α* (DA1) mRNA injected embryos, 1 day after injection with live, heat killed or ΔRD1 Mm. Data shown are mean ± SEM, n = 74–108 cells from 15 embryos combined from 3 independent experiments.

To address this hypothesis we used a genetic approach to modulate tyrosine nitration levels in neutrophils [46]. Hypoxia inducible factor 1α (HIF-1α) stabilization was shown to be involved in leukocyte activation and host defense in murine and zebrafish models of inflammation and infection [71]–[74]. We have previously reported that stabilized Hif-1α signaling upregulated neutrophil nitrotyrosine levels in zebrafish embryos in the absence of infection [46]. We investigated what happened to this elevated level of anti-nitrotyrosine at 1 dpi after injection with live, heat killed, or ΔRD1 Mm into embryos with stabilized Hif-1α signaling using dominant active *hif-1αb*. At this stage of infection the infection is systemic and granuloma structures have yet to aggregate. We observed that exposure to heat killed Mm further increased tyrosine nitration levels of neutrophils, while infection with living wild type or ΔRD1 Mm significantly decreased tyrosine nitration levels compared to mock-injected controls (figure 7A and B). These data show that recognition of heat-killed Mm increased host NO output, even when tyrosine nitration of neutrophils was genetically increased by Hif-1α stabilization prior to infection. Furthermore, these data demonstrate that live Mm lowered the levels of this nitrosative defense mechanism, independently of the RD1 virulence locus.

## Discussion

Pathogenic mycobacteria have evolved as metabolically flexible bacteria that can sense and adapt to the continuously changing host environment during infection [75]. Mtb is continuously exposed to endogenous ROS produced by normal aerobic respiration, but also exogenous ROS and RNS generated by the host immune system in the granuloma [75], [76]. The presence of macrophages and neutrophils expressing NOS proteins has been demonstrated in mammalian and human Mtb granulomas [77]. What is currently unclear is how the production of RNS in leukocytes is instigated upon mycobacterial infection and what in vivo effect, if any, this has on mycobacterial pathogenesis. The zebrafish Mm model has become a well-accepted model for human TB pathogenesis [41], [43]. In this study we utilized the zebrafish Mm infection model to investigate the RNS host-response to infection and observed that TLR-MyD88 signaling was required for the induction of the nitrosative defense mechanism. Furthermore, we found that live Mm are capable of attenuating the nitrosative response generated by neutrophils during the innate immune response, a phenomenon not observed with heat-killed Mm.

It has previously been difficult to quantify nitration output in leukocytes, however using an anti-nitrotyrosine antibody staining protocol we can accurately measure the nitration output of neutrophils and compare it to the Mpx-dependent basal levels. Our data demonstrate a robust upregulation of tyrosine nitration levels in neutrophils after Mm infection in a zebrafish host, shown by anti-nitrotyrosine antibody staining, alongside DAF FM-DA and anti-iNOS staining. Although the brightest fluorescence was observed intracellularly, low-level extracellular ‘speckled’ staining was also observed, both with and without infection, which may indicate extracellular tissue nitration as observed in another fish model [44], or may be due to non-specific binding of antibody. As the vast majority of nitrotyrosine staining was observed in neutrophils we used intracellular levels as our readout of tyrosine nitration. The presence of heat killed, live or ΔRD1 Mm was sufficient to increase the host neutrophil tyrosine nitration levels 12 hours after injection of Mm, demonstrating that the recognition of mycobacterial PAMPs, rather than virulence or viability of the bacteria, is responsible for this effect. It has been known for a number of years that recognition of the cell wall component lipoarabinomannan (LAM) by leukocytes resulted in iNOS-NO production directly [78], or via induction of *iNOS* expression regulating pro-inflammatory cytokines [79], [80] and here we show upregulation in an in vivo model of infection that corroborates these findings. We have previously shown that although macrophages are the key phagocyte type responsible for initial phagocytosis of Mm [62] and are able to produce ROS and upregulate iNOS in response to infection [81], [82], it was the population of neutrophils that showed a robust increase in tyrosine nitration at the systemic level [46]. Neutrophils have been previously implicated in protection against Mycobacteria in the early granuloma by oxidative killing, but our data indicate that at the onset of infection neutrophils have the capacity to produce an RNS response against Mycobacteria [83]. This observation indicates the possibility of cytokine signaling, released from infected macrophages, causing neutrophils to systemically upregulate their iNOS program. As it is likely a signal of infected macrophages to neutrophil that leads to this upregulation, we postulated that Il8 may be the cytokine responsible for this, as it is a key macrophage to neutrophil attraction signal that can also act systemically. Upon infection, macrophages phagocytose Mm rapidly (within the first 30 minutes post infection), therefore infected macrophages have the potential to interact with a large number of neutrophils over the course of the first 24 hpi. Furthermore, the receptor for Il-8, Cxcr2, has been shown to be specifically expressed by neutrophils in the zebrafish embryo [67]. Despite this, blocking Il8/Cxcr2 signaling both pharmacologically and genetically caused no change in the nitrosative response by neutrophils to Mm infection indicating that the Il-8/Cxcr2 axis is not responsible for the increase in uninfected neutrophil nitrotyrosine. The range of chemokine signaling in the zebrafish host response has yet to be fully elucidated and identification of the signal that is responsible for activation of neutrophils requires further study and understanding of these receptor families. Further mechanistic evidence that pro-inflammatory cytokine release is integral to this response is the observation that the increase in tyrosine nitration was absent in *myd88^−/−^* embryos infected with Mm. As previously shown, these MyD88-deficient embryos display a significantly reduced cytokine response (van der Vaart et al., 2013). The absence of infection-induced tyrosine nitration in *myd88−/−* embryos is therefore consistent with the mycobacterial cell wall containing lipoproteins and lipoglycans serving as TLR ligands [84] that stimulate neutrophil iNOS output, either directly or via macrophage signaling. The observation that tyrosine nitration could also be induced by heat-killed Mm further supports that TLR recognition of Mm is required to initiate this nitrosative defense mechanism in zebrafish neutrophils.

Neutrophils, both bystanders and those infected, have increased nitrotyrosine/iNOS levels at 1 dpi after infection with Mm and Salmonella in the zebrafish embryo [46], [85]. However, it was interesting to note from our previous work that neither morpholino knockdown of iNOS, encoded by the *nos2a* gene in zebrafish, nor pan-NOS or iNOS specific inhibitor treatment resulted in significantly increased Mm bacterial burdens [46]. By upregulating the tyrosine nitration levels of neutrophils prior to infection using dominant active *hif-1αb* we have previously demonstrated an associated decrease in bacterial burden [46]. These data indicate that the natural NO response is not an effective host defense mechanism against Mm infection, however, when levels of neutrophil NO are artificially boosted to be high at the point of infection, then there is an increased anti-microbial effect, targeting the mycobacteria before they have time to adapt.

Emerging evidence suggests that mycobacteria are able to manipulate cells of the host to allow for intracellular growth. This has been shown in vivo during *Mycobacterium leprae* infection, in which the mycobacteria can transcriptionally reprogramme their host cells, Schwann cells, into a stem-cell like state [86]. Mtb are able to sense and react to reactive oxygen/nitrogen species in their surrounding environment, for example, the Mtb WhiB4 protein can sense O_2_ and NO levels and activate the oxidative stress response, which maintains the redox balance and modulates virulence [87]. Exposure of Mtb to H_2_O_2_ or NO initiates a robust transcriptional response, including the expression of cellular repair mechanisms and upregulation of genes that perform ROS and RNS scavenging functions [35], [36]. We observed a reduction in nitrotyrosine staining in the center of granulomas of Mm-infected zebrafish embryos. While we cannot rule out that this decrease in staining is due to dead or degranulating neutrophils these data are suggestive of Mm being able to counteract neutrophil tyrosine nitration levels before they are able to have a depletory effect on bacterial load. We therefore went on to investigate this possibility at pre-granuloma stages using the dominant active *hif-1αb* increase in neutrophil NO response as a model to investigate whether this was a bacterial-driven response. Attenuated and heat-killed strains do not replicate and form granulomas at the same rate as live Mm, therefore comparing granulomas from different Mm types at 3 or 4 dpi would be technically difficult. The dominant active *hif-1αb* model allowed investigation at 1 dpi, a stage at which infection is still systemic before granulomas have begun to aggregate. In the Hif-1α-stabilized model we observed that tyrosine nitration attenuation was independent of the RD1 virulence locus, required for survival and replication inside macrophages, but that this effect was not caused by heat-killed bacteria which increased the nitrotyrosine levels further. These findings are in contrast to the induction of tyrosine nitration, which is induced by ΔRD1 and heatkilled Mm suggesting alternative regulation of induction and subsequent attenuation of tyrosine nitration. Together, these data suggest that Mm infection initially induces increased NO production in neutrophils and that this response is attenuated during the course of infection by a bacterial virulence mechanism. A bacterial protein potentially involved in regulating this oxidative stress response is OxyR [88]. Additionally, mycobacteria can directly protect themselves against oxidative stress via AhpC and MsrA and B, by catalyzing the breakdown of peroxynitrite or protecting Mtb against its detrimental effects [89]–[91]. Recently it has been demonstrated in vivo that Mm and Mtb have specialized lipids on their membrane that block recruitment of microbicidal macrophages that produce RNS, but not permissive macrophages that do not produce RNS [82]. Our data are not inconsistent with these findings and further demonstrate the complex underlying mechanisms by which Mycobacteria evade the host RNS response. Virulence mechanisms of Mtb and Mm have evolved to specifically counteract the oxidative defenses of their natural human and zebrafish hosts, which may partly explain why Mtb is more susceptible to RNS production in the non-natural mouse model of TB disease [78], [92].

The search for new anti-TB therapies has intensified with the increasing occurrence of Mtb strains that are multi-drug resistant [1], [2]. The attenuation of RNS activity described here may be directly linked to multi-drug resistance, since resistance of clinical Mtb strains to first-line antibiotics was demonstrated to be associated with decreased susceptibility to NO [93]. Our previous data indicate that Mm are more susceptible to increased host nitrosative defenses at early stages of infection when induced by stabilized Hif-1α, before they have the opportunity to sense and manipulate the host immune response [46]. Our new findings show that the nitrosative host defense is dependent on TLR signaling and are suggestive of Mm manipulation of the host response leading to a down regulation of the nitrosative host defense. Taken together these data have furthered the understanding of the host RNS response to mycobacterial infection and identifies iNOS as a potential host-derived drug target to increase the host RNS response to combat infection, a strategy that should bypass multi-drug resistance mechanisms of the bacteria.

## Supporting Information

Figure S1 Anti-nitrotyrosine predominantly labels neutrophils in granulomas. Example fluorescent micrographs of anti-nitrotyrosine staining performed on 4 dpi granuloma structures after infection with Mm. The staining colocalized, in the main, with the mpx:GFP fluorescence of neutrophils. Two brightly stained example cells are indicated by the white arrow heads.
(TIF)

Figure S2 Heat killed *M. marinum* does not grow on plates. The injection dose of live, heat killed or ΔRD1 Mm were plated for CFU counts. Heat-killed Mm did not grow on appropriate media after incubation for a week.
(TIF)

Figure S3 Nitrotyrosine levels in progression granulomas are lower when co-localized with bacteria. (A) Example fluorescence confocal micrographs of anti-nitrotyrosine staining performed on 5 dpi granuloma structures in wild type embryos infected with Mm. A merged image of extended focus is shown in the top left panel while the signal Z-plane used for the measurements is shown in the top right panel. (B) Gray values of the bacterial and anti-nitrotyrosine fluorescence signals measured along a straight line through the center of the granuloma (see white lines in (A)), intensity of the fluorescent signal was measured using ImageJ. Numbered peaks in the graphs correspond to numbered patches of tyrosine nitration in (A).
(TIF)
