# Supplementary figures and images for: Mycobacteria Counteract a TLR-Mediated Nitrosative Defense Mechanism in a Zebrafish Infection Model
Source: PLoS One. 2014 Jun 26;9(6):e100928. doi: 10.1371/journal.pone.0100928 (PMC4072692; doi:10.1371/journal.pone.0100928)

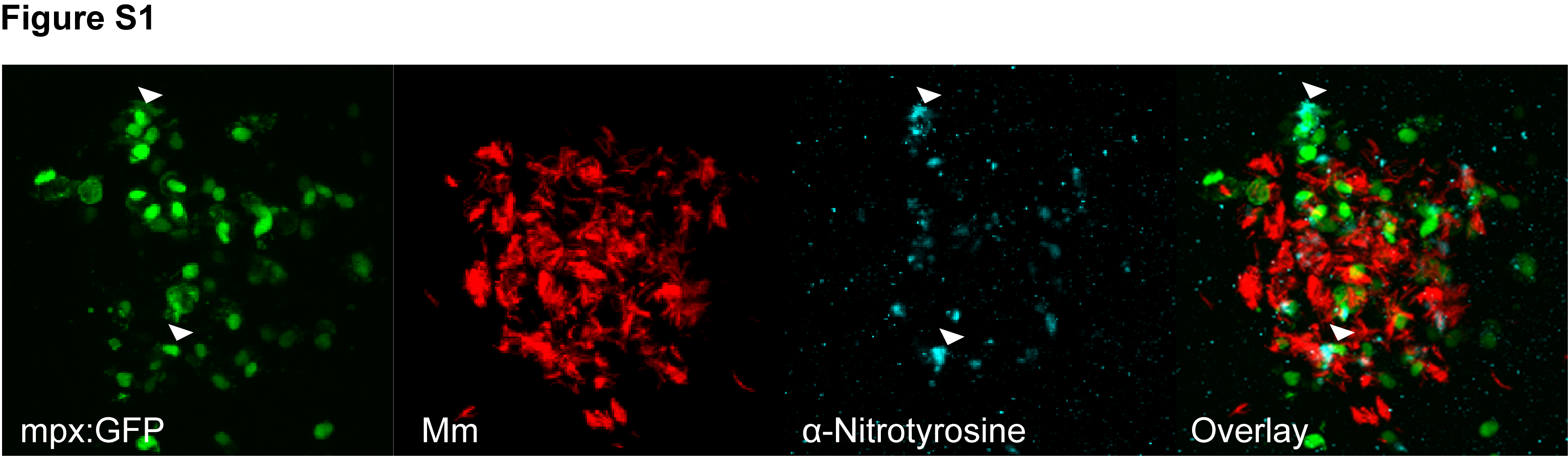

Supplement: Figure S1 — Anti-nitrotyrosine predominantly labels neutrophils in granulomas. Example fluorescent micrographs of anti-nitrotyrosine staining performed on 4 dpi granuloma structures after infection with Mm. The staining colocalized, in the main, with the mpx:GFP fluorescence of neutrophils. Two brightly stained example cells are indicated by the white arrow heads. (TIF) [file pone.0100928.s001.tif]

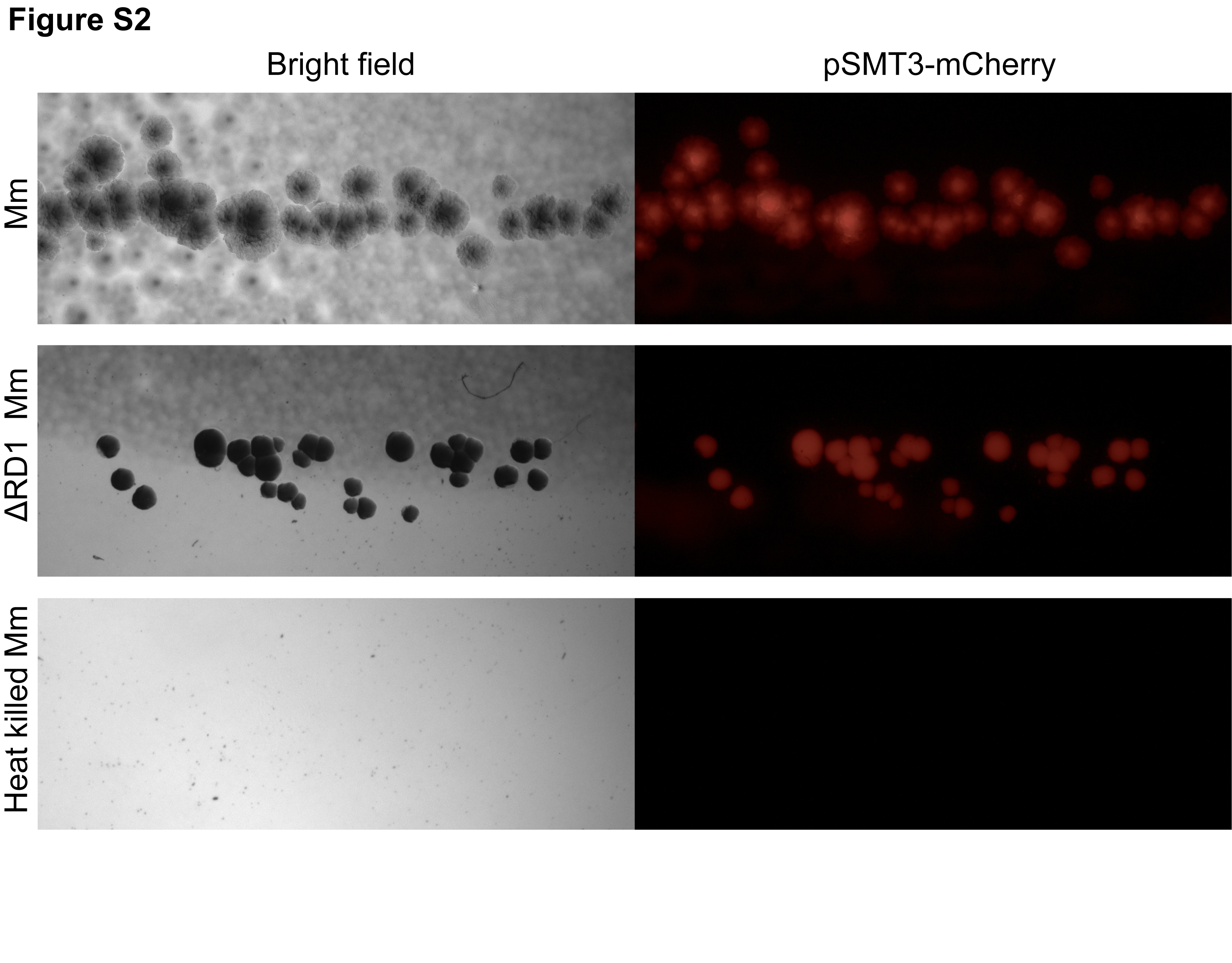

Supplement: Figure S2 — Heat killed M. marinum does not grow on plates. The injection dose of live, heat killed or ΔRD1 Mm were plated for CFU counts. Heat-killed Mm did not grow on appropriate media after incubation for a week. (TIF) [file pone.0100928.s002.tif]

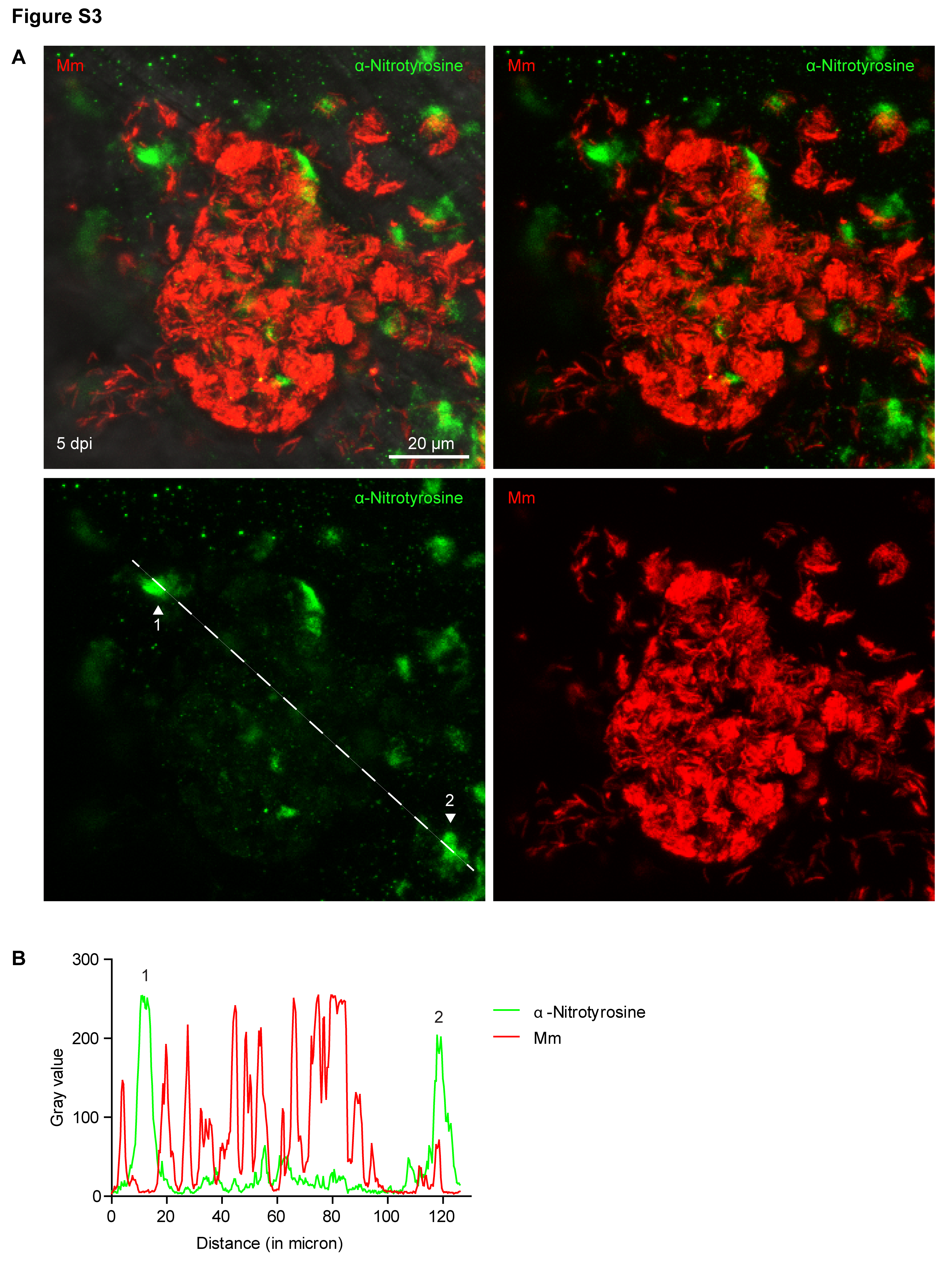

Supplement: Figure S3 — Nitrotyrosine levels in progression granulomas are lower when co-localized with bacteria. (A) Example fluorescence confocal micrographs of anti-nitrotyrosine staining performed on 5 dpi granuloma structures in wild type embryos infected with Mm. A merged image of extended focus is shown in the top left panel while the signal Z-plane used for the measurements is shown in the top right panel. (B) Gray values of the bacterial and anti-nitrotyrosine fluorescence signals measured along a straight line through the center of the granuloma (see white lines in (A)), intensity of the fluorescent signal was measured using ImageJ. Numbered peaks in the graphs correspond to numbered patches of tyrosine nitration in (A). (TIF) [file pone.0100928.s003.tif]
